# Links between Gestures and Multisensory Processing: Individual Differences Suggest a Compensation Mechanism

**DOI:** 10.3389/fpsyg.2017.01828

**Published:** 2017-10-16

**Authors:** Simon B. Schmalenbach, Jutta Billino, Tilo Kircher, Bianca M. van Kemenade, Benjamin Straube

**Affiliations:** ^1^Department of Psychiatry and Psychotherapy, Philipps-Universität Marburg, Marburg, Germany; ^2^Department of Psychology, Justus Liebig University Giessen, Giessen, Germany

**Keywords:** gesture, communication, individual differences, action perception, multisensory processing

## Abstract

Speech-associated gestures represent an important communication modality. However, individual differences in the production and perception of gestures are not well understood so far. We hypothesized that the perception of multisensory action consequences might play a crucial role. Verbal communication involves continuous calibration of audio–visual information produced by the speakers. The effective production and perception of gestures supporting this process could depend on the given capacities to perceive multisensory information accurately. We explored the association between the production and perception of gestures and the monitoring of multisensory action consequences in a sample of 31 participants. We applied a recently introduced gesture scale to assess self-reported gesture production and perception in everyday life situations. In the perceptual experiment, we presented unimodal (visual) and bimodal (visual and auditory) sensory outcomes with various delays after a self-initiated (active) or externally generated (passive) button press. Participants had to report whether they detected a delay between the button press and the visual stimulus. We derived psychometric functions for each condition and determined points of subjective equality, reflecting detection thresholds for delays. Results support a robust link between gesture scores and detection thresholds. Individuals with higher detection thresholds (lower performance) reported more frequent gesture production and perception and furthermore profited more from multisensory information in the experimental task. We propose that our findings indicate a compensational function of multisensory processing as a basis for individual differences in both action outcome monitoring and gesture production and perception in everyday life situations.

## Introduction

Speech-associated gestures provide an important attribute of everyday communication. Although their role in communicating has been explored in detail (Goldin-Meadow and Alibali, 2013), individual differences in the production and perception of gestures are still barely understood. The role of gestures in communication has been found to be beneficial for the listener as well as for the speaker. Listeners’ comprehension of speech is improved when accompanied by gestures and gestures improve learning of speech content (Kelly, 2001; Valenzeno et al., 2003; Straube et al., 2009). They thus can be considered as a fundamental additional communication modality that complements speech, i.e., the auditory modality. Furthermore, gestures can compensate for constraints in auditory perception, e.g., in noisy contexts or in patients with hearing problems (Obermeier et al., 2012). However, individuals have been reported to differ substantially in their production and perception of gestures in everyday life (Hostetter and Alibali, 2007; Nagels et al., 2015). Insights into which mechanisms contribute to these individual differences are just beginning to emerge. Cognitive skills have been found to be associated with gesture production (Hostetter and Alibali, 2007; Chu et al., 2014), but the role of perceptual processes has not been considered so far.

When talking about individual differences in speech associated gestures we also have to consider the origin of speech-associated gestures. One of the most prominent models to describe the origin of gestures is the gesture-as-simulated-action (GSA) model (Hostetter and Alibali, 2008). This model postulates that simulations of actions and perceptual consequences naturally go with speech production. Simulating and planning actions as well as just observing actions share neural correlates with actual performing of actions (Jeannerod, 2001). Most of these simulated actions stay covert, while some become overt. According to the GSA model, gestures are overt simulated actions. Simulation of actions and their perceptual consequences are known to support understanding and learning of actions (Pascual-Leone et al., 1995; Gallese and Goldman, 1998; Jeannerod, 2001). Congruent to these general findings, it has been shown that the use of gestures fosters, e.g., problem solving and multitasking (Goldin-Meadow et al., 2001; Chu and Kita, 2011). Furthermore, there is accumulating evidence that the use of gestures also provides a compensational mechanism when, e.g., cognitive resources for mastering a given task are insufficient (Hostetter and Alibali, 2007; Chu et al., 2014). In addition Holle et al. (2012) showed a compensational role for speech-associated gestures in ambiguous speech and furthermore described a bimodal enhancement of speech-associated gestures (Holle et al., 2010). In summary, it can be stated that the compensational role of speech-associated gestures seems to be widely accepted.

Simulation of actions involves the simulation of motoric, sensomotoric, as well as auditory and visual action consequences. Therefore, action-simulation crucially involves the integration and matching of multisensory information. This can be assumed to be functionally associated with the production and perception of gestures. Undeniably there are substantial differences between self-generated (active) and externally generated (passive) movements. When self-performing an action the perception of motor-sensory outcomes is compared to predictions generated for the action consequences. Those predictions are continuously generated to attain a correct and efficient processing of sensory signals. Generating predictions for action consequences is an efficient way to reduce surprise and to distinguish between one’s own actions and causes of the environment. If the action is not self-performed, i.e., happens passively to the body, much weaker predictions for action consequences are generated and reactions to external stimuli are delayed compared to self-initiated actions (Wolpert and Flanagan, 2001; Cullen, 2004). The underlying theoretical framework explaining this mechanism is called the *forward model* and was first described by von Holst and Mittelstaedt (1950). According to their model, our central nervous system uses a copy of the motor command, a so called efference copy, to predict sensory effects of our own actions (von Holst and Mittelstaedt, 1950; Wolpert et al., 1995; Wolpert and Miall, 1996). The predicted sensory outcome is then compared with the actual sensory feedback and resulting matches or mismatches can be used to suppress or enhance processing of resulting stimuli, thereby guiding our behavior. Predictive mechanisms have primarily been investigated unimodally, in particular in the visual (Leube et al., 2003; Farrer et al., 2008; Hoover and Harris, 2012), auditory (Ford et al., 2005; Wang et al., 2014), and tactile modality (Blakemore et al., 1998). Only recently van Kemenade et al. (2016) explored predictive mechanisms under bimodal conditions showing a facilitating effect. These findings were congruent with previous seminal evidence for facilitation of perception when multisensory information is integrated (McDonald et al., 2000; Ernst and Banks, 2002; Ernst and Bulthoff, 2004). Most importantly, an advantage under multimodal conditions appears highly relevant for gesture production and perception in everyday life communication involving continuous calibration of multisensory information. In this context, gestures as predictive actions (Pouw and Hostetter, 2016) and their multisensory representation might explain the facilitative effects in everyday situations.

Considering the GSA model and the framework for multisensory integration, we were interested in whether a link between speech-associated gestures production and perception and the monitoring of multisensory action consequences can be observed. We explored individual differences in gesture production and perception using the recently introduced Brief Assessment of Gesture (BAG) scale that allows assessment of self-reported gesture production and perception in everyday communication (Nagels et al., 2015). In parallel we determined individual perceptual abilities to detect the temporal mismatch of unimodal as well as bimodal action consequences in both active and passive action conditions (see van Kemenade et al., 2016). As in the study by van Kemenade et al. (2016), participants performed button presses, which led to the presentation of unimodal or bimodal audio–visual stimuli, presented with a variable delay. In active task modalities, participants pressed the button themselves, whereas in passive conditions, the button was pulled down by an electromagnet. van Kemenade et al. (2016) showed an enhanced performance through bimodal stimuli which was in line with previous findings indicating a facilitating effect under bimodal conditions (McDonald et al., 2000; Ernst and Banks, 2002; Ernst and Bulthoff, 2004). However, individual differences variability in the capacities to match sensory information to action consequences has not been investigated. Since both the GSA model and multisensory integration share overlapping elements, e.g., the simulation of actions, we speculated that potential individual differences in the applied task might be linked to individual differences in speech-associated gestures production and perception. As stated above the compensational role of speech-associated gestures production and perception has been reported repeatedly. We therefore hypothesized a negative relationship between self-reported gesture production and perception and the perception of action consequences, assuming a compensational mechanism for speech-associated gestures. Given the multisensory character of gestures in verbal communication, we moreover expected a particularly pronounced link to bimodal facilitation.

## Materials and Methods

### Participants

The participants were 34 students enrolled at the Philipps University of Marburg (19 male, age range 18–30 years, *M* = 24.3, *SD* = 3.3). All participants were right-handed and had normal or corrected-to-normal vision. The study was approved by the local ethics committee and participants gave written informed consent. Results from a subsample of 24 subjects in the perceptual task have been published recently without considering individual differences (van Kemenade et al., 2016).

### Measures and Procedures

Gesture production and perception were measured by the BAG scale (Nagels et al., 2015). This self-report questionnaire comprises 12 items which have to be judged on a 5-point Likert scale (1 = not agree, 5 = fully agree). Exemplary items are “I usually gesture a lot when I talk to make myself understood better.” or “I like talking to people who gesture a lot when they talk.” The BAG scale has been shown to offer robust psychometric properties and provides a convenient measure of gesture usage, though ultimate validation by objective measures is still pending. The questionnaire covers both gesture perception and production. Since both gesture perception and production potentially involve multisensory processes, we derived the average score across all items as a comprehensive measure of gesture production and perception. A higher score on the BAG scale means the participant reported to use more gestures and to pay more attention to gestures during conversation compared to a participant with a lower score.

Perception of action consequences was assessed by a recently introduced uni- and multisensory temporal mismatch paradigm (see van Kemenade et al., 2016). Details can be found in the original publication. The experiment was conducted in a quiet, dimly lit room. Participants sat behind a 60 Hz computer screen with a total viewing distance of 54 cm. A chin rest was used to stabilize participants’ head position. Participants were asked to place their right hand on a button pad, with their right index finger touching the button. The button pad was placed under a black box during the experiment so participants were not able to see their right hand.

In the experiment participants performed button presses, either self-initiated (active) or externally initiated by a custom-made device (passive). Button presses gave rise to either a visual dot stimulus (unimodal condition) or a visual–auditory stimulus combining a dot and a tone (bimodal condition). The stimuli occurred either immediately after the button press or after a variable delay. Unimodal stimuli were presented for 1 s. In bimodal conditions the first appearing stimulus was presented for 1 s, the second stimulus disappeared at the same time as the first stimulus did. Both stimuli thus disappeared at the same time, regardless of their individual onset time. Participants had to decide whether there was a delay between button press and visual stimulus presentation or whether both events occurred synchronously. In the unimodal condition, six defined delay periods were used (0, 83, 167, 250, 333, or 417 ms). In the bimodal condition, the same sample of delay periods was used for the visual modality and the auditory stimulus was induced with one of three delays (0, 167, or 417 ms). We presented 10 trials for each delay, thus leading to 60 active and 60 passive unimodal visual trials. Furthermore, for the bimodal condition, we presented 60 active and 60 passive trials with a non-delayed auditory modality, 60 active and 60 passive trials with the auditory stimulus delayed by 167 ms, and 60 active and 60 passive trials with the auditory stimulus delayed by 417 ms. To balance out the potential effects of stimulus congruency, we also added congruent bimodal trials for the delays of 83, 250, and 333 ms, with 10 trials for each delay and both active and passive conditions. With these additional 60 trials, the total number of trials added up to 540 per participant. Prior to the actual experiment, participants could press the button several times to see delayed (417 ms) and non-delayed feedback. Furthermore, the button was pulled down automatically a few times to show delayed (417 ms) and non-delayed feedback after a passive button press. Then, to become familiar with the paradigm, they completed a short training (20 trials) with the same procedure as the main experiment, during which feedback (“correct” or “incorrect”) about their performance was provided. In the main experiment, no feedback was given. In the main experiment each trial had the same course of action: a trial started with an intertrial interval (1, 1.5, or 2 s) with a fixation cross, after which a cue appeared in the form of the outline of a square (3.2° visual angle), surrounding the fixation cross. In active conditions the square indicated that from now on, participants could press the button with their right index finger. They were instructed to wait with their button press for at least 700 ms after the appearance of the square and of course as long as they wanted. This was done to elicit a well prepared, self-initiated button press, rather than an automatic action as a reflex to the cue (Rohde and Ernst, 2013). If participants pressed the button too early, the text “Too early” was presented on the computer screen and the trial had to be repeated. When the button was pressed correctly at least 700 ms after the square appeared, the sensory feedback was presented. In passive blocks, the procedure was very similar to active blocks. Here, the same square indicated that from now on, the button could be pulled down automatically. The time between square appearance and passive button press was jittered (0.5–3.5 s). After offset of the stimuli, a 500 ms interval with a fixation cross followed. After this, the question “Delay? Yes/No” was presented on the screen. Participants were given 4 s to answer. Their left hand was therefore placed on a keyboard. With their left middle finger they pressed a button on the keyboard for answering “Yes, there was a delay” and with their left index finger they pressed a button on the keyboard for “No, there was no delay.”

The experiment allowed measurement of the ability to detect delays of action consequences in active and passive conditions. We determined detection thresholds for temporal mismatch of action outcomes. Psychometric functions were fitted to the data using the psignifit toolbox in Matlab (Frund et al., 2011). Points of subjective equality (PSE) give the ability to detect delays, lower values reflecting better performance. PSE reflects the detection threshold at which 50% of delays are detected. We had to exclude three participants from further analyses because they failed to reach a performance of at least 50% for the longest delay so that it was not possible to determine thresholds.

### Statistical Analysis

We fitted psychometric function curves to the data with the Psignifit toolbox in Matlab (Frund et al., 2011). After this we were able to determine participants’ PSE. We then performed 2 (unimodal vs. bimodal feedback) × 2 (active vs. passive action conditions) ANOVAs with repeated measures to analyze main effects of feedback type and action condition. We performed *t*-tests to test our hypotheses mentioned above.

## Results

Evaluating the BAG scale we derived an average gesture processing score across all items for each participant. The score could range between 1 and 5, higher scores indicating higher use of gestures in communication situations. Our participants reported clear speech-associated gestures use, but showed also considerable individual differences (*M* = 3.57, *SD* = 0.53).

**FIGURE 1:**
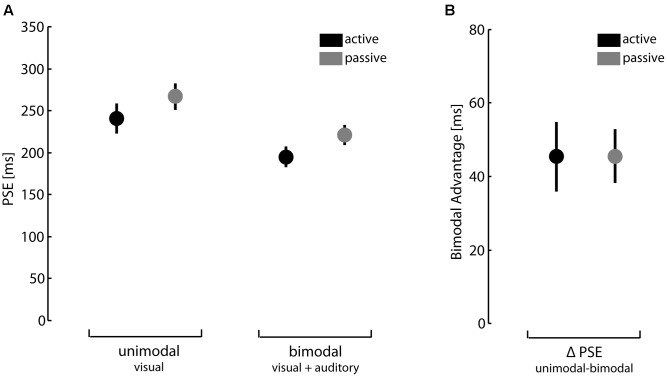
Perception of action consequences. **(A)** Points of subjective equality (PSE) for unimodal and bimodal feedback under active and passive action conditions. **(B)** Difference between PSEs for unimodal and bimodal feedback under active and passive action conditions. Error bars depict standard errors of mean.

The PSE in the perceptual task are illustrated in **Figure 1A**. We analyzed performance data by 2 (unimodal vs. bimodal feedback) × 2 (active vs. passive action conditions) ANOVA with repeated measures. Congruent with earlier findings using this task (see van Kemenade et al., 2016), results showed significant main effects of feedback type and action condition, *F*(1,30) = 43.30, *p* < 0.01, $\eta_{p}^{2}$ = 0.59 and *F*(1,30) = 15.90, *p* < 0.01, $\eta_{p}^{2}$ = 0.35, respectively. We found no significant interaction effect between feedback type and action condition, *F*(1,30) < 0.01, *p* = 0.99, $\eta_{p}^{2}$ < 0.01. In summary, participants were better in detecting a temporal mismatch of action outcomes when given bimodal feedback compared to unimodal feedback. Furthermore, performance was better under active action conditions than under passive action conditions. Since we were in particular interested in the bimodal advantage, we calculated for each participant the difference between unimodal and bimodal PSEs. Results are depicted in **Figure 1B**. The bimodal advantage did not differ under active and passive action conditions, *t*(30) = -0.012, *p* = 0.99.

**FIGURE 2:**
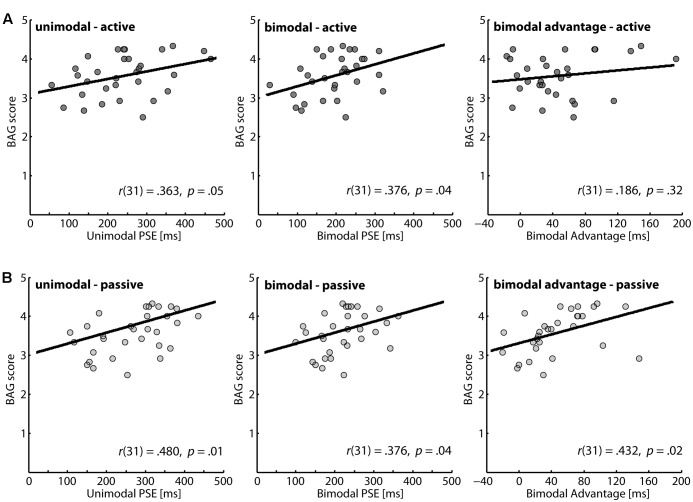
Correlation between brief assessment of gestures (BAG) scores and the perception of action consequences. A higher PSE value indicates a lower performance in the perceptual task. **(A)** Active condition: the first two panels illustrate data for unimodal and bimodal feedback, respectively; the left panel gives data for the bimodal advantage. **(B)** Passive condition: the first two panels illustrate data for unimodal and bimodal feedback, respectively; the left panel gives data for the bimodal advantage.

Our main interest was the association between gesture score and the perception of action outcomes. The correlations of the BAG scores with the different performance parameters in our perceptual task are presented in **Figure 2**. For all parameters positive correlations were determined, and except for the bimodal advantage in the active action condition all correlation coefficients reached significance. Thus, we found robust evidence that higher detection thresholds for a temporal mismatch of action outcomes are associated with more pronounced gesture score.

**FIGURE 3:**
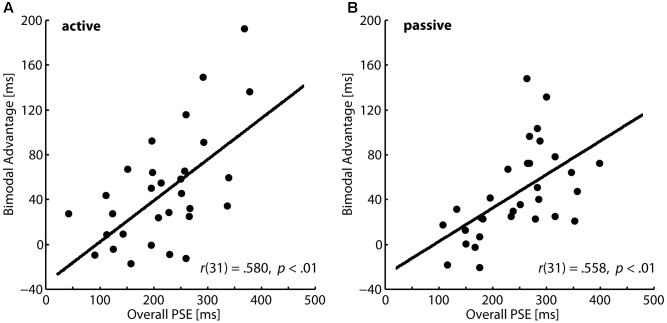
Correlations between average PSEs and the bimodal advantages in the temporal mismatch task. **(A)** Active condition and **(B)** passive condition.

The correlational results indicated that lower capacities to perceive action consequences outcome might be compensated by an enhanced production and perception of gestures during communication. In order to explore compensational mechanisms further, we investigated whether the bimodal advantage in the temporal mismatch task depends on the overall perceptual performance. We averaged participants’ PSE values in the unimodal and the bimodal conditions and correlated this general perceptual measure with the bimodal advantage. In the active action condition as well as in the passive action condition we found significant positive correlations as illustrated in **Figures 3A,B**, respectively. Participants with higher PSEs, i.e., lower perceptual performance, show a significantly higher bimodal advantage. This pattern also points to a compensational function of multisensory information.

## Discussion

The aim of the present study was to investigate the relationship between self-reported gesture production and perception in everyday life and the monitoring of one’s own action consequences in a delay detection task. Here we provided new evidence about this relationship, suggesting that lower performance in detecting delays in sensory consequences of active and passive movements is related to increased bimodal advantage in this task and a more pronounced gesture production and perception in everyday life situations. Detection thresholds for delays in action consequences were identified in a perceptual task in which participants were asked to detect an outcome delay after a self-generated or externally generated button press. Results for the perceptual task showed significantly lower thresholds in bimodal conditions, in active as well as in passive action conditions. Extending previous findings regarding bimodal advantages in a delay detection task (van Kemenade et al., 2016; Straube et al., 2017), we showed that participants with general lower performance, i.e., higher PSEs, profited more in bimodal conditions. Participants’ gesture production and perception were measured on a self-report gesture scale, which correlated positively with performance in the perceptual task, meaning participants with lower performance in the perceptual task scored higher in the questionnaire. Moreover, participants with a higher bimodal advantage in the passive condition reported a more frequent production and perception of gestures in everyday life situations. These data suggest that a lower performance in perceiving delayed action consequences is related to an increased bimodal advantage in this task and more pronounced role for gestures in everyday life situations. Thus, people with lower perceptual abilities demonstrated a compensatory use of multimodal information which is related to individual differences in production and perception of gestures.

Although little is known about the role of perception of action consequences in gesture behavior, there are some findings in line with our results. It has for example been found that gestures are likely to be used to lighten the cognitive load when pressure is put on the internal computational system by cognitive task demands (Goldin-Meadow et al., 2001). Further experiments showed that individuals with low working memory capacity performed worse in a working memory task when gestures were forbidden than people with high working memory capacity (Marstaller and Burianova, 2013; Pouw et al., 2014). Moreover, Chu et al. (2014) analyzed individual differences in spatial working memory capacity, spatial transformation ability and conceptualization ability, and showed that poorer performance in each of these variables was associated with higher production of representational gestures in gesture elicitation tasks. In addition, Pouw and Hostetter (2016) pointed out the predictive function of gestures qualifying them a genuinely cognitive resource. These findings support the idea that gestures play a compensatory role in individuals with low perceptual or cognitive abilities.

Regarding the perceptual aspects of gestures there is also some evidence in line with our findings. For example, individuals are more likely to attend to gestures when communication is hindered, e.g., by noise (Obermeier et al., 2012). Furthermore, comprehension and memory are improved when speech is accompanied by gestures (Kelly, 2001; Valenzeno et al., 2003; Straube et al., 2009; Pouw et al., 2014; Nagels et al., 2015). Pouw et al. (2014) proposed that the cognitive system is more likely to employ an externally supported problem solving strategy, if cheaply available, when the costs of an internal computation are high. This is either induced externally (e.g., by high cognitive demand of the task) or internally (e.g., by lower cognitive abilities). A possible explanation of our results might be that participants with lower perceptual abilities rely more on multisensory information in everyday communication.

Our data support an association between lower perceptual task performance and higher social communicative behavior in terms of gesture production and perception. Since gesture production and perception were assessed by a self-report questionnaire, conclusions are certainly limited by the subjective character of measurement. For our analyses, we used the general score of the BAG, since we consider gesture production and perception as inseparably linked to multisensory processing in everyday communication. The association between actual gesture production and perception and multisensory capacities should be explored in more detail. For our next experiment in this field we will create an online version of the BAG scale that allows quick screening of a large number of participants. Then, we will invite participants with a high BAG score (>95th percentile) or with a low BAG score (<5th percentile), respectively, to take part in our experiments. Further experimental insights could be gained by manipulating reliability of multisensory information and investigating the effects on gesture production and perception in real communication situations. Additionally, other parameters, e.g., social skills could be investigated in future studies regarding their possible link to perception of action consequences. Through this we hope to confirm our previous findings and further investigate the relationship between speech-associated gestures and multisensory processing. However, the BAG scale has been introduced as an efficient assessment of gesture perception and production in everyday communication and is suited to assess individual differences (Nagels et al., 2015). Whether the use of more objective measures for gesture production and perception would qualify our findings awaits clarification, but we expect similar results. We found first evidence that individuals with lower performance in monitoring of active and passive action consequences show enhanced self-reported gesture production and perception, possibly as a compensational mechanism. Our results suggest a compensatory role of bimodal action outcomes also in the perceptual task *per se*, as indicated by a significant correlation between general performance and the bimodal advantage. Thus, participants with lower performance in the perceptual task benefited more in bimodal conditions, which speaks in favor of compensation. Most importantly, we found a significant positive correlation between the bimodal advantage in the passive condition and the BAG score. Participants with a higher advantage in the passive bimodal task scored higher in the questionnaire. This result fits nicely with our idea that multisensory integration on its own provides an additional source of information, which can be used to compensate at different levels of processing. However, there are some open questions arising from our results.

First, it seems notable that the correlation between the BAG scores and the bimodal advantage is only present in the passive condition. A possible explanation might be that the association is attenuated by the efference copy present in the active condition; the link might become functional only when the efference copy is absent. It should be noted here, that a link with the bimodal advantage may be different from the general effects; after all, the BAG score did correlate with both unimodal and bimodal performance in active and passive conditions. It remains unclear why the efference copy would have an attenuating effect only on the bimodal advantage. The underlying mechanisms thus have to be clarified in future studies. Furthermore, the role of performance capacities for interpreting our results should be explored in more detail. Our analysis shows significantly lower thresholds in active conditions compared to passive ones. This is in line with previous experiments indicating an enhancing effect of the efference copy (Shimada et al., 2010; Hoover and Harris, 2012; van Kemenade et al., 2016). It can be consequently assumed that detecting delays was more difficult in the passive, than in the active condition. As described above, participants with lower performance in cognitive tasks have been shown to report more gesture in a gesture elicitation task (Chu et al., 2014). This might suggest that participants with lower ability in detecting delays found the passive tasks even harder and reported higher gesture production and perception in everyday communication. Thus, our findings should be considered as a tentative evidence for a link between the monitoring of multisensory action consequences and self-reported production and perception of speech-associated gestures.

In summary, we have shown that a lower performance in perceiving action consequences is linked to more pronounced role of gestures in everyday life situations as well as increased bimodal advantage. We propose compensatory multisensory processing, related to gesture perception and production in everyday life situations, in individuals who show lower abilities to perceive the consequences of their actions accurately.

## Ethics Statement

This study was carried out in accordance with the recommendations of the ethics committee of the medicine faculty of Philipps-University Marburg with written informed consent from all subjects. All subjects gave written informed consent in accordance with the Declaration of Helsinki. The protocol was approved by the ethics committee of the medicine faculty of Philipps-University Marburg.

## Author Contributions

BvK, TK, and BS designed the study. SS collected and analyzed the data. SS, JB, TK, BvK, and BS interpreted the data and wrote the paper.

## Conflict of Interest Statement

The authors declare that the research was conducted in the absence of any commercial or financial relationships that could be construed as a potential conflict of interest.
